# Evaluation of differentially expressed genes identified in keratoconus

**Published:** 2009-11-28

**Authors:** Ji-Eun Lee, Boo Sup Oum, Hee Young Choi, Seung Uk Lee, Jong Soo Lee

**Affiliations:** 1 The Department of Ophthalmology, College of Medicine, Pusan National University, Pusan, Korea; 2 Medical Research Institute, Pusan National University Hospital, Pusan, Korea

## Abstract

**Purpose:**

To identify the differentially expressed genes (DEGs) in the human keratocytes in keratoconus.

**Methods:**

Total RNA extracted from cultured corneal stromal fibroblasts from normal and keratoconic corneas were used for the synthesis of cDNA. DEGs were screened by an annealing control primer^TM^-based PCR method using GeneFishing^™^ DEG kits. The differentially expressed bands were sequenced and analyzed. The genes identified were further evaluated by reverse transcriptase PCR and quantitative real-time PCR.

**Results:**

Overexpression of bone morphogenetic protein 4 (*BMP4*), cofilin 1 (*CFL1*), and JAW1-related protein (*MRVI1*) and underexpression of actin, alpha 2 (*ACTA2*), gene rich cluster, and C 10 gene (*GRCC10*), tissue inhibitor of metalloproteinase 3 (*TIMP3*), tissue inhibitor of metalloproteinase 1 (*TIMP1*), and somatostatin receptor 1 (*SSTR1*) were verified, and these results were confirmed by reverse transcriptase PCR and quantitative real-time PCR.

**Conclusions:**

Eight genes were identified to be differentially expressed in keratoconus and related with apoptosis, the cytoskeleton, wound healing, and nerve fibers. The genes identified may be involved in the mechanism underlying stromal thinning; thus, they could be important and deserve further investigation.

## Introduction

Keratoconus is characterized by thinning of the corneal stroma, but its pathological mechanism has not been fully elucidated. Clinical studies have suggested that the disease has a high incidence among long-term users of contact lenses, often attacks those who have a history of rubbing their eyes, and is related to atopy of the eye [1-4]. Therefore, it is believed that the long-term damage to and stimulation of the corneal epithelium play a key role in the pathogenesis of keratoconus. Furthermore, it has been proven that when the corneal epithelium is damaged, surrounding stromal keratocytes disappear due to apoptosis, and that an imbalance between cell death and proliferation is involved in the pathological mechanism of keratoconus. The apoptosis of keratocytes plays an important role in the corneal thinning in keratoconus [5]. Hence, it is valuable to investigate the genes of the keratocytes that are involved in the thinning of the cornea because the it is important to grasp the fundamental pathogenesis in order to understand and treat the disease of keratoconus. Even though there have been studies on the morphologic or ultrastructural differences between normal and keratoconic cornea, there are few reports on their differentially expressed genes (DEGs) [6-9]. Kim et al. [6-8] compared the differential gene expression in the keratoconic and normal corneal epithelium using microarray focusing on the epithelium; their study had the advantage of early detection using the simple gene expression analysis of the scraped-off epithelium. Ha et al. [9] used keratoconic and normal cultured keratocyte and complementary DNA (cDNA) microarrays to find apoptosis-related genes, and concentrated on the importance of programmed cell death as having a crucial role in the corneal thinning in keratoconus. In this study, we investigated the DEGs between normal and keratoconic cultured corneal stromal fibroblasts; the results could be used as preliminary data for further study of the molecular mechanism underlying keratoconus.

## Methods

### Culture of human keratocytes

This study was conducted in accordance with the tenets of the Declaration of Helsinki. Normal corneas were obtained using human donor corneas that had been discarded before transplantation due to mild endothelial polymorphous dystrophy, and keratoconic corneas came from patients with keratoconus found at keratoplasty with informed consent. This study used five samples each of normal and keratoconic corneas. Stromal explants were prepared by removing the epithelium and endothelium, followed by culturing in Dulbecco's Modified Eagle Medium (DMEM; Gibco, BRL, NY), that contains 10% fetal bovine serum (FBS; Gibco), 100 units/ml penicillin (Gibco), and 100 mg/ml streptomycin (Gibco). Culture medium was replaced every two to three days. When the cells reached confluence, the culture medium was completely removed and the cells were washed with Dulbecco's Phosphate-Buffered Saline (D-PBS; Gibco) and detached enzymatically with 0.25% trypsin–0.02% EDTA (Gibco). For genetic analysis, cultured corneal stromal fibroblasts of the third to fourth passage were collected and kept at -70 °C until used for experiments.

### Messenger RNA isolation

Using the RNeasy^®^ mini kit (QIAGEN Inc., Valencia, CA), all RNA was extracted from the cultured corneal stromal fibroblasts of normal and keratoconic corneas that were cultured for genetic analysis. First, the buffer was added to the cell pellets and they were homogenized for 30 s using a rotor-stator homogenizer. After adding the ethanol and mixing well by pipetting, the top layer solution that contained RNA was applied to an RNeasy^®^ minicolumn (QIAGEN Inc.) placed in a collection tube. After repeating the processes of centrifugation, washing, and adding the buffer, the remaining solution was centrifuged and RNase-free water was used to elute the RNA. The separated messenger RNA (mRNA) was measured and checked by electrophoresis.

### cDNA Synthesis

The mRNA extracted from cultured corneal stromal fibroblasts was employed for the synthesis of first-strand cDNA by reverse transcriptase, as described by Hwang et al. [10]. Reverse transcription was performed for 1.5 h at 42 °C in a final reaction volume of 20 μl containing 3 μg of the purified total RNA, 4 μl of 5× reaction buffer (Promega, Madison, WI), 5 μl of deoxyribonucleotide triphosphate (each 2 mmol), 2 μl of 10 μmol cDNA synthesis primer deoxythiamine annealing control primer 1 (dT-ACP1; Table 1), 0.5 μl of RNasin^®^ RNase Inhibitor (40 U/μl; Promega), and 1 μl of Moloney murine leukemia virus reverse transcriptase (200 U/μl; Promega). First-strand cDNA was diluted by the addition of 80 μl of ultra-purified water for the GeneFishing^™^ PCR, and stored at -20 °C until use.

**Table 1:** Primer sequence used in cDNA synthesis and ACP^™^-based PCR.

**Use**	**Primer name**	**Sequence**
cDNA synthesis primer	dT-ACP1	5’-CTGTGAATGCTGCGACTACGATIIIII(T) 18-3’
Reverse primer	dT-ACP2	5’-CTGTGAATGCTGCGACTACGATIIIII(T) 15-3’
Arbitrary primer	ACP12	5’-GTCTACCAGGCATTCGCTTCATIIIIIGCCAGGAAGA-3’
(forward primer)	ACP17	5’-GTCTACCAGGCATTCGCTTCATIIIIICCATGAAGAT-3’
	ACP21	5’-GTCTACCAGGCATTCGCTTCATIIIIIGGCCCTGGAT-3’
	ACP22	5’-GTCTACCAGGCATTCGCTTCATIIIIIAGTTCGTGGG-3’
	ACP25	5’-GTCTACCAGGCATTCGCTTCATIIIIICCTACGCCAC-3’
	ACP27	5’-GTCTACCAGGCATTCGCTTCATIIIIIATTATTTATA-3’
	ACP28	5’-GTCTACCAGGCATTCGCTTCATIIIIICCTTGAGCGC-3’
	ACP31	5’-GTCTACCAGGCATTCGCTTCATIIIIIAGGAAAAAAG-3’

ACP, annealing control primer; The polydeoxyinosine [poly(dI)] linkers are underlined. In the table, "I" represents deoxyinosine.

### Annealing control primer^™^-based GeneFishing^™^ PCR

DEGs were screened by the annealing control primer (ACP)^™^-based PCR method using the GeneFishing^™^ DEG kits (Seegene, Seoul, South Korea) [11]. The GeneFishing^™^ PCR technique involved an ACP^™^ system that had a unique tripartite structure in that its distinct 3’-end target core sequence and 5’-end non-target universal sequence portions were separated by a regulator, it used primers that annealed specifically to the template, and it allowed only genuine products to be amplified; this process that eliminated false positive results. Second-strand cDNA synthesis and subsequent PCR amplification were conducted in a single tube. Briefly, second-strand cDNA synthesis was conducted at 50 °C (low stringency) during one cycle of first-stage PCR in a final reaction volume of 49.5 μl containing 3–5 μl (about 50 ng) of diluted first-strand cDNA, 5 μl of 10× PCR buffer plus Mg (Roche Applied Science, Mannheim, Germany), 5 μl of dNTP (each 2 mM), 1 μl of 10 μM dT-ACP2, and 1 μl of 10 μM arbitrary primer preheated to 94 °C (Table 1). The tube containing the reaction mixture was held at 94 ºC, while 0.5 μl of Taq DNA Polymerase (5 U/μl; Roche Applied Science) was added to the reaction mixture. The PCR protocol for second-strand synthesis was one cycle at 94 ºC for 1 min, followed by 50 ºC for 3 min, and 72 ºC for 1 min. After the completion of second-strand DNA synthesis, 40 cycles were performed. Each cycle involved denaturation at 94 ºC for 40 s, annealing at 65 ºC for 40 s, extension at 72 ºC for 40 s, and a final extension at 72 ºC to complete the reaction. The amplified PCR products were separated in 2% agarose gel stained with ethidium bromide.

### Cloning and sequencing

The differentially expressed bands were extracted from the gel using the GENCLEAN^®^ II Kit (Q-BIO gene, Carlsbad, CA), and directly cloned into a TOPO TA^®^ cloning vector (Invitrogen, Karlsruhe, Germany) according to the manufacturer’s instructions. The cloned plasmids were sequenced with an ABI PRISM^®^ 3100 Genetic Analyzer (Applied Biosystems, Foster City, CA). Complete sequences were analyzed by searching for similarities using the Basic Local Alignment Search Tool (BLAST) search program at the National Center for Biotechnology Information GenBank [12].

### Reverse transcriptase-polymerase chain reaction and quantitative real-time PCR confirmation

The differential expression of DEGs was confirmed by reverse transcriptase–polymerase chain reaction (RT-PCR) using each gene-specific primer pair. The primer sets and annealing temperatures for eight genes are shown in Table 2. The cDNA was amplified using primers derived from the sequence of the DEGs and glyceraldehydes 3-phosphate dehydrogenase (*GAPDH*) as a control reference. The PCR reaction was conducted in a final reaction volume of 20 μl containing 2–4 μl (about 50 ng) of diluted first-strand cDNA, 1 μl of primer 5’ (10 μM), 1 μl of primer 3’ (10 μM), and 10 μl of 2× Master Mix^®^ (Seegene). The PCR amplification protocol was an initial 3 min denaturation at 94 ºC, followed by 20–25 cycles of 94 ºC for 40 s, 60 ºC for 40 s, 72 ºC for 40 s, and a 5 min final extension at 72 ºC. The amplified PCR products were separated in 2% agarose gel stained with ethidium bromide.

**Table 2:** Characteristics and sequence-specific primers used for RT-PCR and quantitative real-time PCR of 8 differentially expressed genes.

**Gene**	**GenBank number**	**Primer sequence (5'-3')**	**Annealing temperature (°C)**	**PCR product size (bp)**
Bone morphogenetic protein 4 (*BMP4*)	NM_130850	F: GGGCACCTCATCACACGAC		
		R: TGGGCACACAACAGGCTTT	60	550
Cofilin 1 (*CFL1*)	BC011005	F: TTCTGCGGCTCTCGGTG		
		R: GCTTCTCTGCCAGGGTG	60	499
JAW1-related protein (*MRVI1*)	XM_001166452	F: TGTGGATCTGAAGAGTCC CCCA		
		R: ACAGTTCAATGCTAGCACACCTG	60	282
Actin, alpha 2 (*ACTA2*)	BC017554	F: CCTCCCTTGAGAAGAGTTACG		
		R: GAGCAGGAAAGTGTTTTAGAA	60	443
Gene rich cluster and C 10 gene (*GRCC10*)	BC009925	F; CGTGGCTCTTTATTCGTGA		
		R: GGGCTTCGTAGGACTTGAC	60	333
Tissue inhibitor of metalloproteinase 3 (*TIMP3*)	NM_000362	F: AAGCGATGTCAGAGGGCG		
		R: AACTGGATGGGCAGCAGG	60	465
Tissue inhibitor of metalloproteinase 1 (*TIMP1*)	NM_003254	F: GTTCCAAGCCTTAGGGGATGC		
		R: TGACGTAGTCAGGTCCACCAC T	60	407
Somatostatin receptor 1 (*SSTR1*)	NM_001049	F: CTGGCTTGTACATAGTAGGCAC		
		R:CAATGGTAACAAGTTAAGAGCACT	60	426

In the table, "F" indicates forward strand and "R" indicates reverse strand.

Quantitative real-time PCR was performed in triplicate in 384-well plates; each 20 μl reaction consisted of 10 μl of SYBR Green Master Mix (Applied Biosystems), 0.5 μl of template (10 ng/μl), and 0.8 μl of 10 pM forward and reverse primers of DEGs and *GAPDH* as control reference (Table 2). The PCR amplification protocol was 50 °C for 2 min and 95 °C for 10 min followed by 40 cycles of 95 °C for 30 s, 60 °C for 30 s, and 72 °C for 30 s. Each of the 384-well quantitative real-time PCR plates included serial dilutions (1, 1/2, 1/4, 1/8, and 1/16) of cDNA, which were used to generate relative standard curves for DEGs and *GAPDH*. The real-time PCR analysis was performed on an Applied Biosystems Prism 7900 Sequence Detection System (Applied Biosystems). Relative quantitation with the data obtaining was performed using the relative standard curve method according to the user’s manual. In the relative standard curve method, one of the experimental samples (keratoconus or normal cornea) can be used as the calibrator. Following this method, in the present study, each relative value was divided by the value of the calibrator and the relative expression was presented as the n-fold difference compared to the calibrator.

## Results

**Figure 1:**
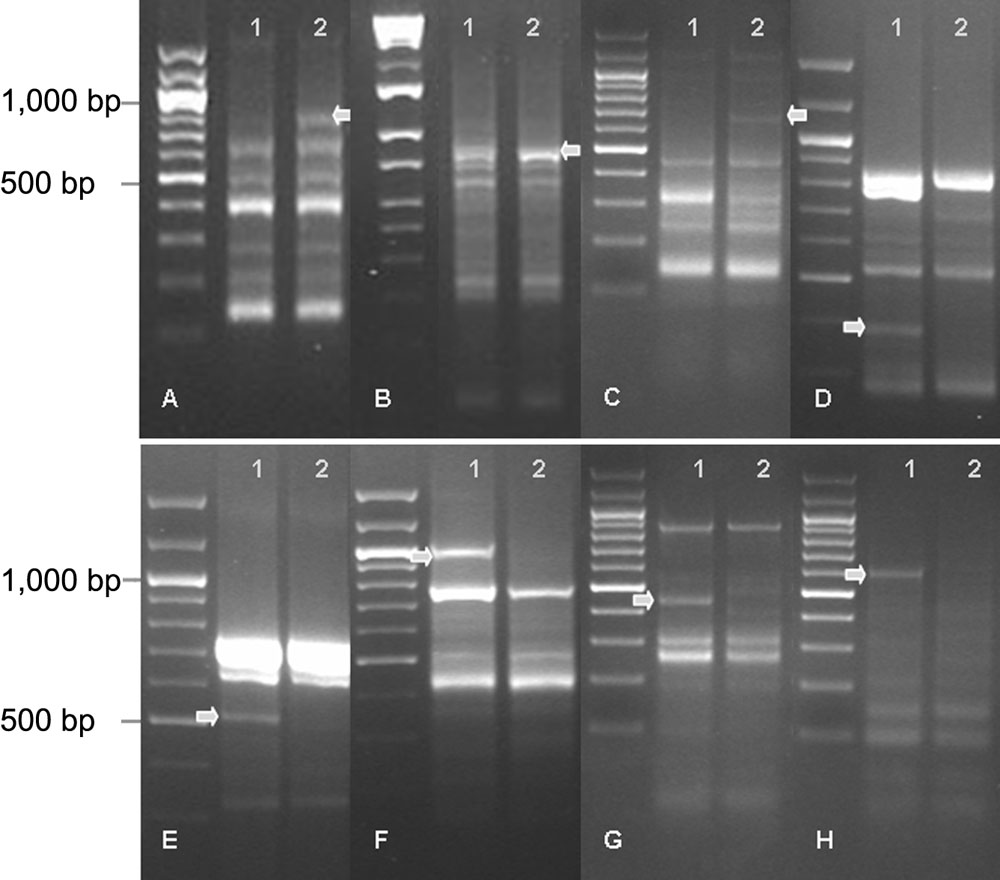
Annealing control primer™–base polymerase chain reaction. Annealing control primer™–base polymerase chain reaction product of eight verified differentially expressed genes shows increased or decreased levels of expression in keratoconus (arrows). Higher levels of expression in keratoconus (column 2) compared with normal cornea (column 1) were found in bone morphogenetic protein 4 (**A**), cofilin 1 (**B**), and JAW1-related protein (**C**), whereas lower levels of expression were found in actin, alpha 2 (**D**), gene rich cluster, and C 10 gene (**E**), tissue inhibitor of metalloproteinase 3 (**F**), tissue inhibitor of metalloproteinase 1 (**G**), and somatostatin receptor 1 (**H**).

The analysis was performed with 5 pairs of normal and keratoconic samples. To identify DEGs, mRNA from normal and keratoconic corneas was extracted and subjected to ACP^™^ analysis using a combination of 120 arbitrary primers and two anchored oligo (dT) primers (dT-ACP 1 and dT-ACP 2). On the basis of the differential expression levels of the mRNA fragments observed on the agarose gels, 8 bands showed the same differential patterns in all 5 pairs of normal and keratoconic samples. Homology searching using the Basic Local Alignment Search Tool revealed their likely identities (Figure 1; Table 2). Three (bone morphogenetic protein 4 [*BMP4*], cofilin 1 [*CFL1*], and JAW1-related protein [*MRVI1*]) were found to be markedly upregulated genes in keratoconus, while the remaining five (actin, alpha 2 [*ACTA2*], gene rich cluster, and C 10 gene [*GRCC10*], tissue inhibitor of metalloproteinase 3 [*TIMP3*], tissue inhibitor of metalloproteinase 1 [*TIMP1*], and somatostatin receptor 1 [*SSTR1*]) were downregulated.

**Figure 2:**
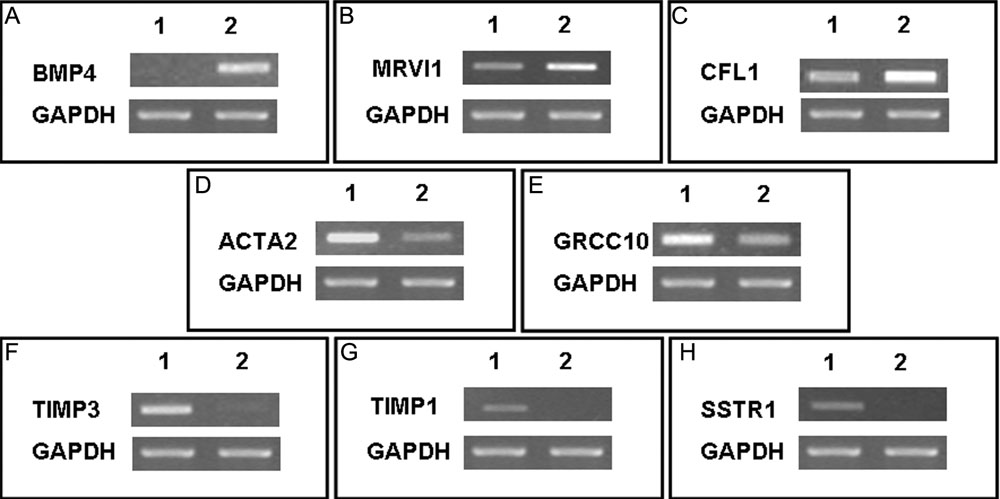
Depicted are reverse transcriptase-polymerase chain reaction products of the eight verified genes and glyceraldehydes 3-phosphate dehydrogenase (*GAPDH*) as a housekeeping gene. Bone morphogenetic protein 4 (*BMP4*; **A**), JAW1-related protein (*MRVI1*; **B**), and cofilin 1 (*CFL1*; **C**) were significantly increased, while actin, alpha 2 (*ACTA2*; **D**), gene rich cluster, and C 10 gene (*GRCC10*; **E**), tissue inhibitor of metalloproteinase 3 (*TIMP 3*; **F**), tissue inhibitor of metalloproteinase 1 (*TIMP1*; **G**), and somatostatin receptor 1 (*SSTR1*; **H**) were significantly decreased in keratoconus (colummn 2) compared with normal cornea (column 1).

**Figure 3:**
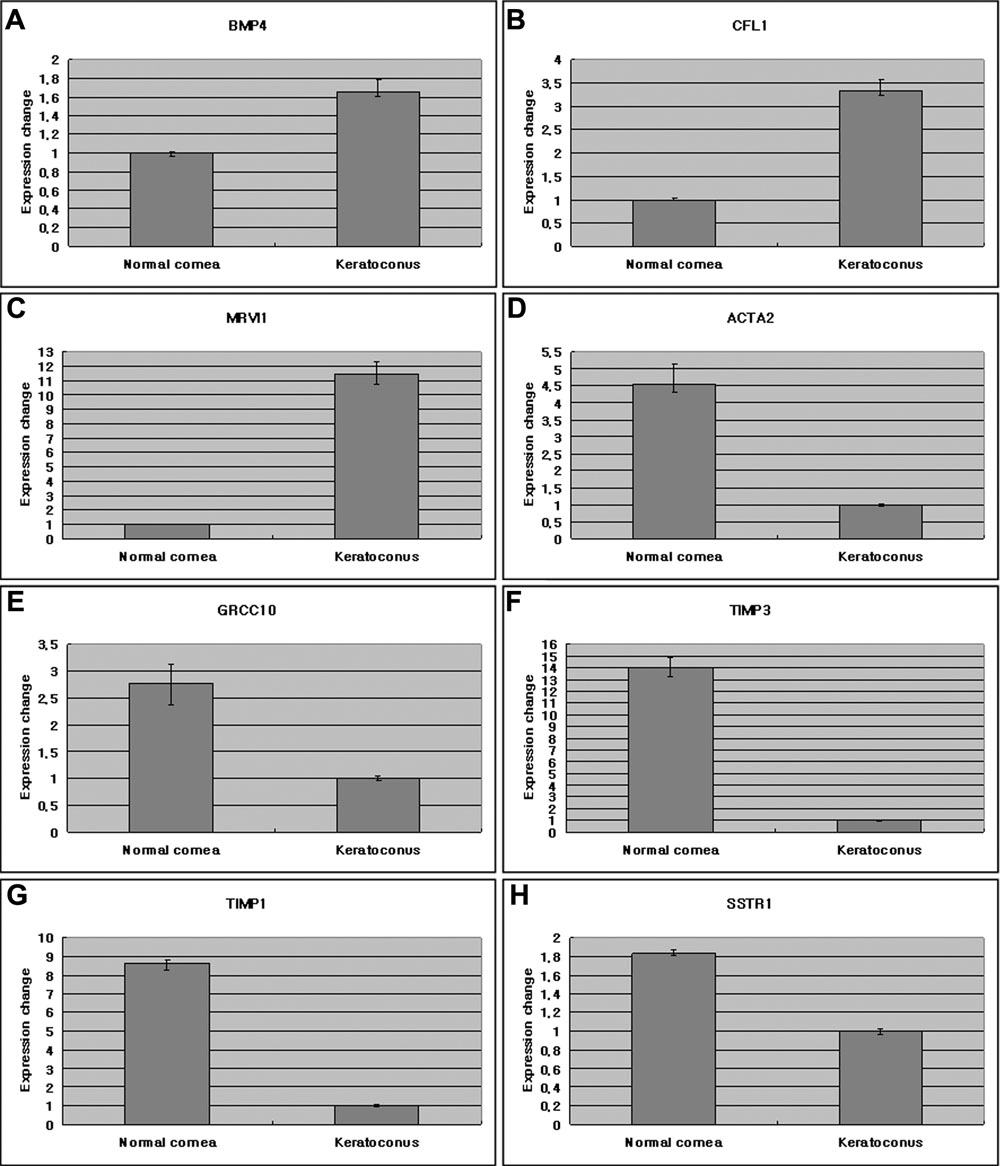
Quantitative real-time polymerase chain reaction. Quantitative real-time polymerase chain reaction of the eight verified genes using glyceraldehydes 3-phosphate dehydrogenase (*GAPDH*) as endogenous control showed that bone morphogenetic protein 4 (*BMP4*; **A**), cofilin 1 (*CFL1*; **B**), JAW1-related protein (*MRVI1*; **C**) were increased by 1.6, 3.3, and 11 fold, respectively, whereas actin, alpha 2 (*ACTA2*; **D**), gene rich cluster, and C 10 gene (*GRCC10*; **E**), tissue inhibitor of metalloproteinase 3 (*TIMP3*; **F**), tissue inhibitor of metalloproteinase 1 (*TIMP1*; **G**), and somatostatin receptor 1 (*SSTR1*; **H**) were decreased by 4.5, 2.7, 14, 8.5, and 1.8 fold, respectively in keratoconus relatively to norma l cornea.

The expression patterns of the DEGs were confirmed by RT-PCR. The RT-PCR assays revealed that in agreement with the ACP^™^ differential display, 8 bands had similar expression patterns (Figure 2). The results of the ACP^™^ analysis were also validated with quantitative real-time PCR. Data obtained after normalization to *GAPDH* showed that, in keratoconus, *BMP4* increased 1.6 fold (Figure 3A), *CFL1* increased 3.3 fold (Figure 3B), *MVI1* increased 11 fold (Figure 3C), *ACTA2* decreased by nearly 4.5 fold (Figure 3D), *GRCC10* decreased by 2.7 fold (Figure 3E), *TIMP3* decreased by nearly 14 fold (Figure 3F), *TIMP1* decreased by 8.5 fold (Figure 3G), and *SSTR1* decreased by 1.8 fold (Figure 3H) relative to a normal cornea. The quantitative real-time PCR assay also revealed similar expression patterns in agreement with the results of ACP^™^ differential display.

## Discussion

Recently, to identify differences in gene expression, microarray analysis with DNA chips has been often used [13]. Using cDNA microarray, it is possible to perform large scale expression surveys to identify the genes whose expression is altered in a disease. However, the change in expression of each gene spot could be variable between sample pairs and within duplicates. This may be due to different incorporation efficiencies of Cy-3 and Cy-5 fluorescence dyes, which may consequently generate variable signal intensity. Therefore, it was necessary to repeat array analysis with different sample pairs in order to find frequently altered genes with a more or less stable pattern of change.

In this study, we employed the new differential display GeneFishing^™^ PCR technique to compare the gene expression in normal and keratoconic corneas [10,11]. Difficulty often arises in identifying a gene responsible for a specialized function during a certain biological stage because the gene is expressed at low levels, whereas the bulk of mRNA transcripts within a cell are highly abundant [14]. To screen DEG transcripts in low concentrations while minimizing the false positive results, it was reasonable to use a PCR-based technique. Moreover, it was possible to detect GeneFishing^™^ technology reaction products easily on ethidium bromide–stained agarose gel. This is supposed to greatly assist studies searching for genes that are expressed differentially in cells under various physiological stages or experimental conditions. However, one of the concerns in this study was the effect of the culturing process; the other was the age and sex matching of sample pairs, which was not possible due to the difficulty in obtaining corneas. With this technique, we identified eight DEGs that are specifically expressed or lacking in keratoconus as compared to normal corneas.

We found that *BMP4* was expressed significantly more highly in keratoconus than in normal corneas. BMP4 is known to mediate apoptosis or alternative developmental fates of neural crest and other types of cells during development [15-17]. BMP4 and its receptor are found in all cell types (epithelial cells, keratocytes, and endothelial cells) of the human cornea [18,19]. Because keratocytes are derived from neural crest, BMP4 stimulates apoptosis in corneal fibroblasts and has a role in mediating keratocyte proliferation and apoptosis in the cornea [15-20]. From the higher expression of BMP4 in keratoconus in this study, it was suggested that BMP4 might play an important role in mediating the apoptosis of keratocytes in keratoconus.

CFL1 is well characterized as an actin depolymerization factor and crucial for many cellular processes, such as cell motility, cell division, and membrane organization. Binding of CFL1 to actin filaments alters the twist of the filament, thereby promoting filament severing and depolymerization. CFL1 is involved in the organization of the cytoskeleton [21,22]. It also has an important function during the initiation phase of apoptosis, which it induces by being translocated to mitochondria during the initial stage of apoptosis [23]. *CFL1* was also a gene whose expression was increased in keratoconus.

*MRVI1*, although no function of this gene is well known, is involved in the control of growth and/or differentiation of hematopoietic cells, and supposed to be a tumor suppressor gene. MRVI1 functions as cofactor in several signaling-transduction systems that affect cellular growth, differentiation, motility, and adhesion [24]. Although it has not been studied in the tissue of cornea, the upregulation of *MRVI1* in keratoconus in this study suggested that MRVI1 might retard the cell cycle of keratocytes and affect the cytoskeleton as well as wound healing. Further studies of MRVI1 involving the cornea should help to define the mechanism of keratoconus related with the *MRVI1* gene.

Meanwhile, ACTA2, which has been known to be involved in the cytoskeleton [25], showed low expression in keratoconus. ACTA2 is a functional marker for a fibroblast subtype that rapidly remodels the extracellular matrix [26]. Keratocytes can become myofibroblasts during corneal injury and wound healing, and the transformation of keratocytes into myofibroblasts is a key point in the healing process [27]. Myofibroblast cells are characterized by alpha–smooth muscle actin expression and have additional differences relative to keratocytes that include increased production of growth factors, collagen, glycosaminoglycans, collagenases, gelatinases, and metalloproteinases associated with remodeling of the collagen and the stroma. *ACTA2* is expressed at the mRNA level within 24 h of injury to the corneal stroma, is expressed at the protein level at day 3, and declines at the mRNA level by day 5. Therefore, *ACTA2* is important for the wound healing process and the cytoskeleton [28,29]. Although alpha smooth muscle actin is normally not expressed in significant levels in keratocytes and the expression of the *ACTA2* gene in the normal cornea could be affected by culture process, it is possible for normal keratocytes to express this gene in case of the potential corneal injury, and further study of *ACTA2* should be undertaken.

*GRCC10* is located at the CD4 locus on human chromosome 12p13 and possesses diverse expression patterns and functions, ranging from signal transduction and glycolysis to regulation of cell proliferation and ubiquitin-dependent proteolysis [30,31]. The underexpression of genes such as *ACTA2* and *GRCC10* in keratoconus was believed to delay the growth of keratocytes that were necessary for the synthesis and maintenance of collagen fibrils and the extracellular matrix.

It is known that there are four types of tissue inhibitor of metalloproteinase (TIMP), and especially TIMP3 has been reported to exist only in interstitial substances. The role of this gene is to control the balance between the destruction and reformation of the corneal tissue by inhibiting the action of matrix metalloproteinase to protect tissues from irreversible destruction and suppress angiogenesis [32]. Because the expression of TIMP3 in keratoconus was decreased relative to the normal cornea, it was suggested that the decreased expression of this gene caused the imbalance between the destruction and reformation of interstitial substances. In agreement with this, it has already been reported that increased matrix metalloproteinase and decreased TIMP levels are related to the formation of keratoconus [33-35].

The level of TIMP1 also decreased significantly in keratoconus compared with the normal cornea. Although keratoconus does not involve extensive scarring or inflammatory infiltrates, considerable degradation of the extracellular matrix occurs [36,37]. A decreased level of TIMP1 increases gelatinase activities and apoptosis, which are found in keratoconus [34,38-41]. Decreased TIMP1 might play a role in the matrix degradation that was the hallmark of keratoconus.

We found that *SSTR1* expression was downregulated in keratoconus. The cell-specific distribution of SSTR1 in normal human eye tissue has been reported, and SSTR1 is found on the cell membrane and cytoplasm of stromal keratocytes [42]. Somatostatin is a ubiquitously distributed cyclic neuropeptide that has diverse biological functions, the most important of which are its neurotransmitter, antisecretory, and antiproliferative functions [43,44]. It has been reported that corneal nerves may play a role in the development and progression of keratoconus, with support for this hypothesis coming from the close proximity of stromal nerve changes to breaks in Bowman’s membrane and the progression of keratoconus observed in a patient with unilateral fifth cranial nerve palsy [45-47]. The significant alteration in corneal nerves in keratoconus has been also documented with a subbasal nerve fiber density 52.7% lower than that in control eyes, and a correlation between nerve density and severity of disease [48], although whether these alterations play a causative role or are secondary manifestations of the underlying disease remains unknown. These studies indicate that the decreased expression of SSTR1 as a receptor for neuropeptides might be involved in the pathophysiology of keratoconus.

It has been a common experience of many investigators to find genes that are identified as differentially expressed but that cannot be verified by other independent means. However, we assessed the expression of eight genes in more detail by employing RT-PCR and real-time PCR, which confirmed the overexpression of *BMP4*, *CFL1*, and *MRVI1*, and the underexpression of *ACTA2*, *GRCC10*, *TIMP3*, *TIMP1*, and *SSTR1* in keratoconus.

We found differential expression of the eight genes in relation to apoptosis, cytoskeleton structure, wound healing, and nerve fiber density in keratoconus using the GeneFishing^™^ PCR technique. The ACP^™^-based strategy was easy, showed a lack of false positives, and yielded reproducible results. Although the detailed function of these genes and their products remain to be determined, they could be important and deserve further investigation, and their identification in this study provides preliminary data for further study of the molecular mechanism underlying keratoconus.
